# Recent Advances on the Positively-Charged Nanofiltration Membranes for Mg^2+^/Li^+^ Separation Through Interfacial Polymerization

**DOI:** 10.3390/nano15130967

**Published:** 2025-06-22

**Authors:** Xinyu Zeng, Chunchun Meng, Zihan Xu, Xinwu Li, Haochen Zhu, Guangming Li

**Affiliations:** State Key Laboratory of Pollution Control and Resources Reuse, Key Laboratory of Yangtze River Water Environment, Ministry of Education, College of Environmental Science and Engineering, Tongji University, 1239 Siping Rd., Shanghai 200092, China; 2331382@tongji.edu.cn (X.Z.); 2410700@tongji.edu.cn (C.M.); 2431360@tongji.edu.cn (Z.X.); 2331404@tongji.edu.cn (X.L.); ligm@tongji.edu.cn (G.L.)

**Keywords:** nanofiltration membrane, interfacial polymerization, positively-charged NF membrane, Mg^2+^/Li^+^ separation

## Abstract

The rapid development of the global energy industry has driven an escalating worldwide demand for lithium resources. As a major lithium source, salt lake brines contain abundant divalent ions that hinder efficient lithium extraction. Compared with conventional lithium recovery technologies, nanofiltration membranes emerge as an energy-efficient and environmentally friendly alternative. Over the past decade, interfacial polymerization has been widely adopted to fabricate nanofiltration membranes for lithium–magnesium separation, with studies confirming the superior performance of positively charged membranes in distinguishing monovalent and divalent cations. This review systematically summarizes recent advancements in positively charged nanofiltration membranes synthesized via interfacial polymerization for lithium–magnesium separation, categorizing the design strategies into five distinct approaches. The correlations between intrinsic membrane structural characteristics and separation performance are critically analyzed. Furthermore, current challenges and future research directions are discussed to provide new perspectives for developing high-performance positively charged composite nanofiltration membranes. This work aims to inspire innovative designs and accelerate the practical implementation of nanofiltration technology in lithium extraction from salt lake brines.

## 1. Introduction

**Figure 1:**
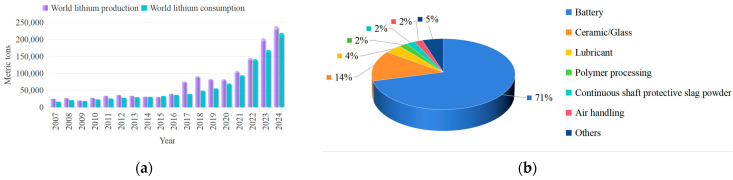
(**a**) Estimated world lithium production and consumption from 2007 to 2024; (**b**) lithium resources application distribution [6].

Renowned as the “white petroleum” of the 21st century due to its low density and lightweight properties combined with high electrochemical activity, lithium has been extensively applied among various fields [1,2,3,4]. The rapid development of the battery industry has particularly driven a dramatic surge in global lithium demand [5]. Figure 1a illustrates the global lithium production and consumption from 2007 to 2024, revealing a sharp increase in both metrics after 2017, with both exceeding 200,000 metric tons by 2024 [6]. However, as shown in Figure 1b, with the widespread promotion and use of new energy vehicles, global lithium resources are mainly applied in the field of lithium-ion batteries [7,8]. Compared with conventional ore extraction methods, lithium recovery from salt lakes has become the mainstream approach due to its generally lower operational costs [9,10].

**Figure 2:**
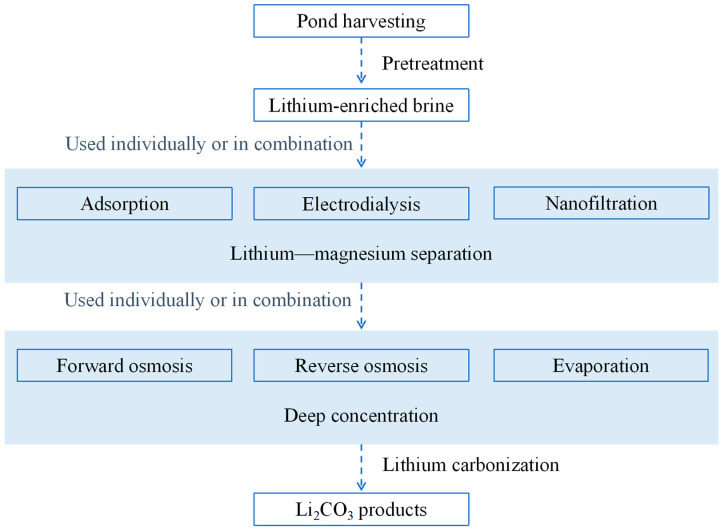
Schematic of lithium extraction system, highlighting the integration of membrane technology in the process chain.

Conventional lithium extraction technologies from salt lakes mainly include precipitation, adsorption, calcination leaching, and solvent extraction [11]. However, the application of these methods are limited by low efficiency, time-consuming processes, and environmental pollution concerns. Membrane separation has emerged as the most promising technology for selective lithium separation due to its environmental friendliness, high separation efficiency, simple processes, and absence of secondary pollution. Nevertheless, coexisting ions in salt lake brines such as Mg^2+^, Ca^2+^, Na^+^, K^+^, Cl^−^, SO_4_^2−^, and CO_3_^2−^ significantly hinder high-purity lithium extraction [7,12]. Particularly, magnesium ions constitute a major obstacle to efficient lithium recovery; they have similar chemical properties and ionic hydrated radii to lithium ions, being 0.428 nm and 0.340 nm, respectively [10]. To address this, especially the critical Li^+^/Mg^2+^ separation, a membrane-based lithium recovery process typically involves three key stages, as illustrated in Figure 2: (1) brine pretreatment (settling, filtration, and softening) to obtain a lithium-enriched brine; (2) critical Mg^2+^/Li^+^ separation using nanofiltration (NF), often integrated with adsorption or electrodialysis; and (3) Concentration of the purified stream via forward osmosis (FO), reverse osmosis (RO), or evaporation, followed by Li_2_CO_3_ precipitation.

NF membranes, positioned between ultrafiltration (UF) and reverse osmosis (RO) in filtration precision, feature pore sizes of 0.5–2 nm [13]. Their separation mechanisms for ions rely on three synergistic effects: (i) size sieving, where solutes larger than membrane pores are retained; (ii) Donnan (electrostatic) exclusion, driven by charge interactions between the membrane surface and ions—repelling co-ions and attracting counter-ions; and (iii) dielectric exclusion, arising from Born energy barriers and image forces in low-dielectric pores [14]. For Mg^2+^/Li^+^ separation, the Donnan effect dominates due to the ions’ similar sizes but divergent charges [15]. Positively charged NF membranes enhance this effect: Their surface cations electrostatically repel Mg^2+^ more strongly than Li^+^, while size sieving amplifies selectivity as hydrated Mg^2+^ is marginally larger [16,17]. This dual mechanism—electrostatic repulsion combined with steric hindrance—enables high Mg^2+^ rejection (>95%) while permitting Li^+^ permeation. Therefore, constructing positively charged membranes by modifying the surface charge characteristics of NF membrane provides a critical solution for lithium extraction from salt lakes with a high Mg^2+^/Li ratio [18].

Since J.M.M Peeters [19] proposed in 1998 that positively charged NF membranes could effectively separate monovalent and divalent cations, Li et al. [20] first applied this to lithium–magnesium ion separation scenarios in 2015. As the most widely used method for NF membrane fabrication, interfacial polymerization (IP)-based NF membranes have dominated the water industry market, and continuous research has been conducted to enhance their separation performance [21]. While recent years have witnessed multiple reviews on NF membranes for Mg^2+^/Li^+^ separation, a systematic summary specifically focusing on positively charged NF membranes remains lacking. Therefore, this review aims to provide a comprehensive and critical overview of recent advancements in positively charged NF membranes fabricated via IP for Mg^2+^/Li^+^ separation. It begins with an analysis of fundamental performance parameters of NF membranes for Mg^2+^/Li^+^ separation, followed by a detailed discussion of design and preparation strategies using IP to engineer positively charged NF membranes. Additionally, factors influencing the Mg^2+^/Li^+^ separation efficiency of these membranes are examined. Finally, the review summarizes current progress, identifies challenges, and proposes future research directions to achieve more significant breakthroughs in Mg^2+^/Li^+^ separation.

## 2. Fundamental Evaluation Parameters for Mg^2+^/Li^+^ Separation Effectiveness

In the Mg^2+^/Li^+^ separation scenario, we refer to the separation performance of NF membranes as two critical performance metrics, namely, Mg^2+^/Li^+^ separation factor (*S_Mg__,Li_*) and pure water permeance (*PWP*). In 2006, Wen and colleagues [22] pioneered the application of a negatively charged NF membrane (Desal-5 DL) for lithium–ion separation from lithium-bearing solutions, marking the inception of membrane-based lithium extraction technology. Although this initial attempt demonstrated a relatively low Mg^2+^/Li^+^ separation factor, it established critical proof-of-concept for subsequent advancements in selective membrane separation processes. Herein, the mentioned separation factor (*S_Mg__,Li_*) was defined as follows:

(1) $$S_{Mg,Li}=\frac{C_{Mg,p}/C_{Li,p}}{C_{Mg,f}/C_{Li,f}}$$

where *C_Mg,p_*, *C_Li,p_*, *C_Mg,f_*, and *C_Li,f_* represent the Mg^2+^ and Li^+^ concentrations on the permeate and feed sides, respectively. The separation efficiency is directly proportional to the *S_Mg__,Li_* value, which means smaller *S_Mg__,Li_* corresponds to superior ion separation performance. Inversely, some scholars employ the opposite notation *S_Li,Mg_* to represent separation factor, as defined in Equation (2), where higher values (*S_Li,Mg_* >1) indicate better separation efficiency.

(2) $$S_{Li,Mg}=\frac{C_{Li,p}/C_{Mg,p}}{C_{Li,f}/C_{Mg,f}}$$

The *PWP* can be calculated as below:

(3) $$PWP=\frac{\Delta V_{P}}{A_{m}\times \Delta t\times \Delta P}$$

where *ΔV_p_* is the permeate volume (L), *A_m_* is the effective area of the membrane (m^2^), *Δt* corresponds to the permeation duration (h), and *ΔP* is the applied transmembrane pressure (bar).

Previous studies have focused on optimizing reaction monomers and conditions to enhance membrane water permeability while maintaining high rejection rates for divalent cations. In this context, rejection selectivity was considered equivalent to permeation selectivity. However, with the growing recognition of the trade-off phenomenon, efforts have been directed toward overcoming the compromise between water permeability and divalent ion rejection in NF membranes [18]. Therefore, in addition to the two parameters mentioned above, the rejection rate (*R*, %) is also an important parameter, representing the proportion of individual ions retained by the membrane. It is calculated using the following formula:

(4) $$R=\left(\begin{array}{c} 1-\frac{C_{p}}{C_{f}} \end{array}\right)\times 100\%$$

where *C_p_* and *C_f_* represent the concentrations of the permeate and feed solutions (ppm), respectively.

## 3. Strategies of Positively-Charged NF Membrane by Interfacial Polymerization

**Figure 3:**
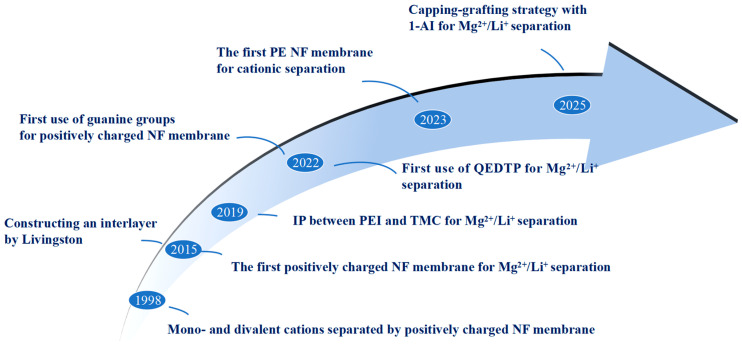
Representative strategies with milestone developments of positively charged NF membrane for Mg^2+^/Li^+^ separation [19,24,25,26,27,28,29,30].

Interfacial polymerization is currently the most widely used method for preparing NF membranes [23], holding a crucial position in fabricating high-performance NF membranes and being extensively applied in various separation scenarios. Typically, two immiscible solvents containing liquid-phase monomers with different functional groups undergo a polycondensation reaction at the interface, forming an ultra-thin, highly cross-linked thin film composite (TFC) membrane with a thickness of several hundred nanometers on the surface of a microporous support membrane. In recent years, positively charged nanofiltration membranes prepared via interfacial polymerization for lithium–magnesium separation have emerged as a research hotspot. Researchers primarily adopt strategies such as monomer selection and optimization, additive incorporation, surface modification, support membrane functionalization, and the introduction of intermediate layers. These novel membranes, designed based on separation mechanisms, significantly enhance selective separation efficiency through precise control of membrane structure, surface charge, and pore size. Representative strategies with milestone developments are chronologically illustrated in Figure 3 below.

### 3.1. Monomer Engineering

**Figure 4:**
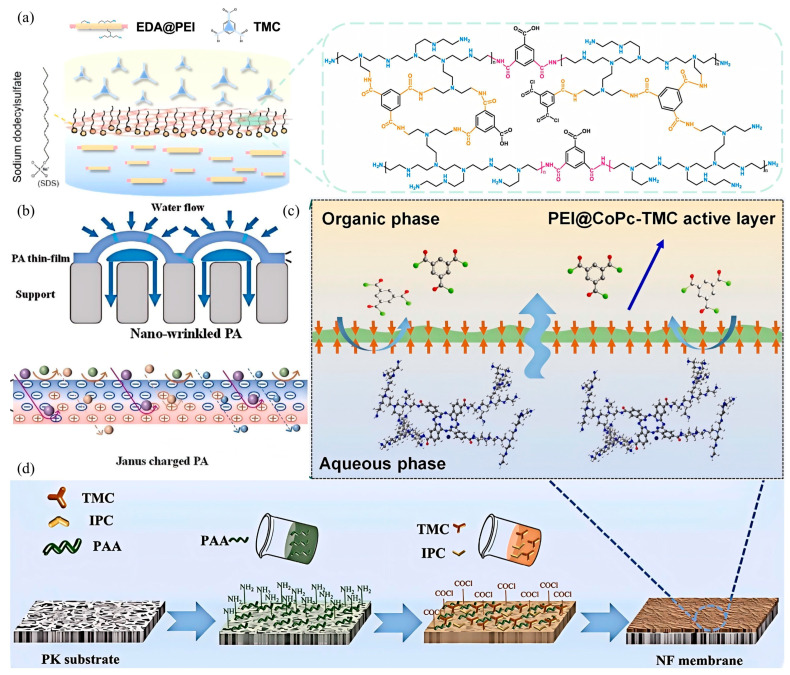
Schematic illustration of preparing lithium–magnesium separation membrane via monomer engineering: (**a**) reaction between EDA@PEI and TMC [31]; (**b**) water pathways and separation of different ions of the prepared PA layer [33]; (**c**) fabrication process of water-soluble PEI@CoPc-modified NF membranes [34]; (**d**) fabrication process of PK/PAA/IPC and PK/PAA/TMC [35].

Since Li et al. [20] in 2015 first fabricated a positively charged hollow-fiber polyamide NF membrane using DAPP and TMC, researchers globally have engineered positively charged NF membranes specifically tailored for targeted lithium extraction. Polyethyleneimine (PEI), as a hyperbranched polymer rich in primary and secondary amines, was used for IP with TMC to target lithium–magnesium separation by Xu et al. [25] in 2019. With no amine groups at either end, PEI cannot react with TMC directly and the PA layer is susceptible to forming defective pores, which leads to a highly compacted polyamide network that restricts water permeability. Subsequently, scholars have modified PEI through graft modification by introducing amino groups at the end of its main chain [31,32]. This modification enables direct connection with TMC, thereby improving the porosity of NF membranes and resulting in a stronger positive charge. For example, ethylenediamine (EDA) was utilized to cap PEI [31], as shown in Figure 4a. With introduced amino groups, the EDA@PEI–TMC modified NF membrane achieved a maximum Mg^2+^ rejection rate of up to 99.21%, with *S_Li__,Mg_* reaching 80.62.

The separation selectivity of NF membranes is primarily determined by steric hindrance and charge interactions [16], while the density and charge characteristics of the PA layer are mainly governed by the inherent properties of reactive monomers [25]. Therefore, the following discussion will focus on these two critical aspects; representative studies are shown in Table 1. Since the positive charge of the PA layer originates from residual amines in polyamine that remain unreacted with acyl chloride, the rejection layer theoretically faces a paradox where strong positive charge conflicts with high crosslinking density. In response, recent studies have focused on designing novel functional monomers to alter membrane structure and separation performance. PIP as an established monomer for IP, and although it lacks sufficient positive charge, novel monomers can be designed based on the PIP skeleton. Lee et al. [36] recently demonstrated that even traditional NF membranes fabricated using PIP/TMC can exhibit a positively charged surface, thereby achieving exceptionally high Li^+^/Mg^2+^ selectivity by simply increasing the PIP concentration. Hence, some researchers have attached quaternary ammonium groups to the intracyclic PIP skeleton via quaternization reactions. The structural extension of PIP-based groups induces a volumetric structural transformation, which facilitates the tunability of the PA membrane’s pore architecture. This endows the newly designed monomers with higher positive charge and free volume, and therefore, the prepared membranes exhibit, simultaneously, enhanced positive charge density, a looser network structure, heterogeneous microporous architecture, increased pore size, narrower pore size distribution, and elevated free volume content in the nanocomposite membranes. Additionally, due to the absence of chlorine-sensitive -NH groups in the formed tertiary amide structures, these membranes also exhibit chlorine stability.

Another key to enhancing NF membrane separation selectivity lies in precisely regulating surface charge density and spatial charge distribution to strengthen ion–membrane interactions [47]. Theoretically, an ideal separation membrane should possess uniform pore sizes. When the pore size is between the molecular dynamic diameters of two solutes, smaller molecules pass through while larger molecules are retained, achieving optimal molecular sieving. However, the precise separation of molecules/ions with similar sizes remains a formidable challenge [48,49]. Recently, Janus membranes with asymmetric properties on both sides have attracted significant attention for enhancing membrane performance [50]. This asymmetric characteristic can provide an intrinsic “internal” driving force at the separation barrier to regulate transport along specific directions [51]. Specifically, Janus-charged NF membranes with asymmetric charge distribution and density can simultaneously intercept both positively and negatively charged ions, or separate ions with different valences [52,53,54]. Studies demonstrate that mixing PEI with PIP as aqueous-phase monomers to prepare Janus-structured NF membranes (dominated by steric hindrance) can generate a PA layer with small pores. During interfacial polymerization, the slower-diffusing PEI accumulates in the lower PA layer while the faster-diffusing PIP aggregates in the upper PA layer, resulting in a Janus-structured PA layer with negative charge on the upper surface and positive charge on the lower surface. The upper Janus PA layer repels divalent anions, while divalent cations permeating to the bottom layer are rejected by the positively charged inner layer. Compared to conventional PA membranes, Janus PA membranes exhibit significantly enhanced rejection rates for both divalent cations and anions. The introduction of PEI not only strengthens the positive charge of the membrane surface but also promotes the formation of nano-wrinkle structures, increasing the membrane surface area. This modification enables the membrane to maintain high water flux while demonstrating superior lithium–magnesium selectivity. Using this approach, modified membranes with a PEI-PIP ratio of 1:4 fabricated by Qi et al. [33] achieved a Mg^2+^/Li^+^ separation factor of 24 (MLR = 20) in simulated brine experiments (shown in Figure 4b), along with a pure water permeability of 16.0 LMH/bar, significantly outperforming the commercial NF-270 membrane (*S_Li,Mg_* = 3.0).

To enhance lithium–magnesium separation selectivity, co-reactive monomers can be introduced into the reaction system to create Li^+^-selective transport channels [46]. In such cases, the sieving effect typically dominates the separation process. Crown ether, as a cyclic ether compound with a repeating -O-CH_2_-CH_2_- structure, exhibits high affinity for lithium ions. Many researchers have functionalized crown ethers by introducing amino groups (-NH_2_), which react with the acyl chloride groups of TMC to form amide bonds, thereby covalently incorporating crown ethers into the PA layer [34,55,56]. For example, He et al. [34] designed a 4-aminophenyl-15-crown-5 incorporated polyamide nanofiltration membrane synthesized via grafting reaction between PEI and (Tetracarboxyphthalocyaninato)cobalt(II), as shown in Figure 4c. With a higher trans-membrane energy barrier and decreased entropic barrier, PEI@4A-B15C5 membrane had a retention rate of 97.3% for magnesium ions.

Overall, modifying the PA layer by designing new monomers, functionalizing PEI, and incorporating co-reactive monomers to enhance crosslinking density and amplify positive charge density on the NF membrane surface has proven effective for performance enhancement. Beyond optimizing aqueous-phase monomers, researchers have developed alternative oil-phase monomers to replace conventional TMC, or introduced co-reactive oil-phase monomers [35,40] (shown in Figure 4d). Although studies on developing positively charged NF membranes by engineering oil-phase monomers remain limited, this approach offers novel insights for designing and selecting innovative oil-phase monomers to construct lithium–magnesium separation membranes with enhanced selectivity and permeability.

### 3.2. Additive Incorporation

In recent years, numerous scholars have enhanced the lithium–magnesium separation performance of NF membranes by incorporating nanomaterials, cyclic molecules, and surfactants into two-phase solutions. Nanomaterials featuring tailored nanopores and nanochannels have been recognized as effective materials for improving membrane selectivity and water flux. Thin-film composite membranes embedded with such nanomaterials are conventionally designated as TFN membranes [57]. Representative studies are shown in Table 2.

**Figure 5:**
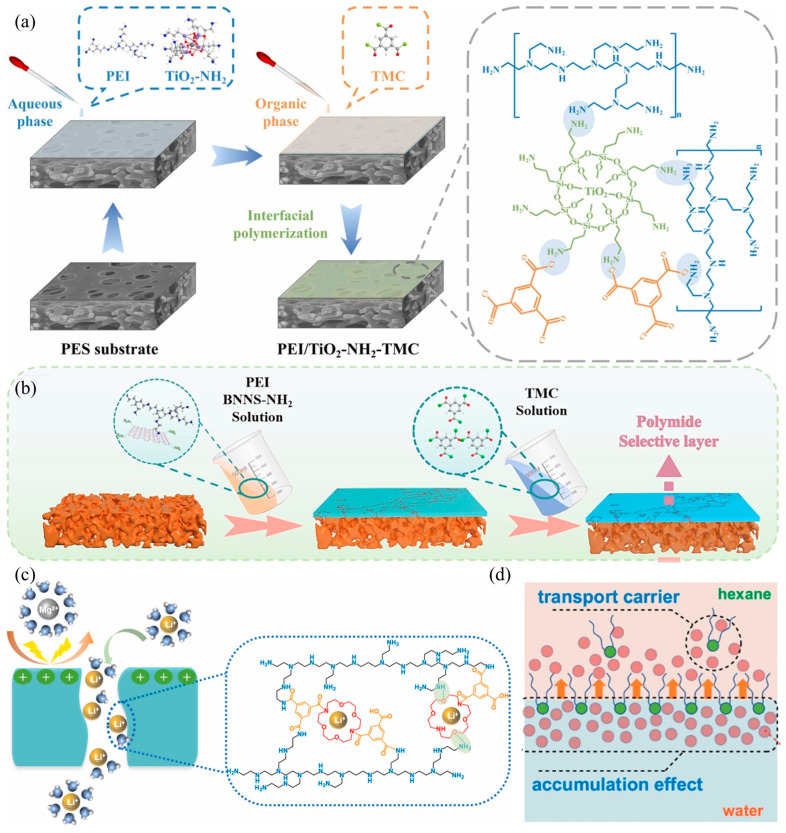
Schematic illustration of preparing lithium–magnesium separation membrane via additive incorporation: (**a**) fabrication process for PEI/TiO_2_-NH_2_-TMC NF membrane [63]; (**b**) IP process of nanofiltration membrane with BNNSs-NH_2_ as the aqueous phase additive [62]; (**c**) cross-linked structure generated from amidation reaction and hydrogen bonding interactions among PEI, DA18C6, and TMC molecules [60]; (**d**) effect of DDP assembly at the oil/water interface on PIP trans-interface diffusion to enhance the homogeneity of the IP reaction [75].

Nanomaterials are typically classified into zero-dimensional (0D), one-dimensional (1D), two-dimensional (2D), and three-dimensional (3D) categories based on their dimensional characteristics. 0D nanomaterials refer to those in which all three spatial dimensions (length, width, and height) are confined within the nanoscale range (1–100 nm), such as graphene quantum dots (GQDs) [73], silica [69], and TiO_2_ [63] (as shown in Figure 5a). However, the instability and aggregation tendency of nanoparticles still leave much room for further improvement towards well-dispersed nanomaterials of good compatibility. A recent study by Yang et al. [74] investigated the influence of nanoparticle properties (e.g., hydrophilicity and porosity) on membrane permeability enhancement mechanisms and water transport dynamics. The research revealed that while nanomaterials can improve membrane water permeability, excessive nanoparticle concentrations induce agglomeration, adversely affecting membrane selectivity. However, in situ nanoparticle formation or surface modification with specialized functional groups has demonstrated the capability to enhance compatibility with PA polymers while effectively suppressing non-selective defect formation. 1D nanomaterials are defined by two dimensions constrained at the nanoscale in three-dimensional space. Carbon nanotubes (CNTs), as representative 1D nanomaterials, provide distinctive water transport pathways through their smooth cylindrical hollow nanostructures and interfacial gaps between CNTs and the polymer matrix. Nevertheless, 1D nanomaterials exhibit agglomeration tendencies comparable to those of their 0D counterparts, whereas optimal membrane permeability enhancement requires aligned CNT orientation parallel to the feed solution flow direction. Xu et al. [71] synthesized a new class of potassium carboxylate functionalized multi-wall CNTs (MWCNTs-COOK) applying an easy-to-operate and environmentally friendly modification method. With the surface more easily ionized in water, MWCNTs-COOK exhibits higher water solubility and can be more uniformly dispersed in water.

Compared to 0D and 1D nanomaterials, two-dimensional (2D) nanomaterials such as carbon nitride, graphene, and metal–organic frameworks (MOFs) demonstrate larger lateral dimensions with atomic-layer thickness. Their unique structural configuration reduces surface energy, thereby mitigating agglomeration tendencies and lowering the crosslinking density of PA layers. Furthermore, these 2D nanomaterials enable the construction of ordered water transport channels through controlled stacking and self-assembly within membranes. Multiple studies have achieved enhanced membrane permeability by incorporating 2D nanomaterials including MOFs [68,72], covalent organic frameworks (COFs) [76], carbon nitride [64,65], and graphene [62]. Wang et al. [64] used a mixed amine solution of PEI and g-C_3_N_5_ to further increase the water permeance and the selectivity for Li^+^/Mg^2+^. The resulting NF membrane with 0.06 wt% g-C_3_N_5_ broke the trade-off effect between selectivity and permeability with an *S_Li,Mg_* value of 18.18 and a pure water flux of 58.59 LMH/bar. As shown in Figure 5b, Li et al. [62] utilized bio-inspired polydopamine (PDA) to modify another analogue to graphene oxide (GO), BNNSs, and introduced it into the long-chain molecules of PEI via intercalation. With uniform dispersion and enhanced affinity between the nanofiller and the polyamide matrix, the optimized membrane exhibited widened spacing between PEI chains and a high permeation flux of 7.62 ± 0.27 LMH/bar. Three-dimensional (3D) nanomaterials refer to bulk materials with nanostructured architectures, such as nano-metals and nano-ceramics. Polyhydroxylated fullerene (PHF) remains the sole 3D nanomaterial reported for TFN membranes in lithium–magnesium separation. Its spherical architecture creates tortuous mass transfer pathways within the polymer matrix, prolonging ion transport duration and amplifying size-sieving effects. Compared to pristine membranes, the optimized membrane exhibited 39.2% enhancement in water flux, increasing from 31.1 to 43.2 LMH/bar [61].

Cyclic molecular architectures such as crown ethers and cyclodextrins (CDs) exhibit distinctive cavity structures that critically function in host–guest chemistry [77,78], simultaneously enhancing lithium–magnesium separation efficiency and optimizing nanofiltration membrane water transport properties. Crown ethers demonstrate selective coordination characteristics toward lithium ions through their cavity configurations, enabling ion-specific transport channels [58,60]. Zha et al. [60] coupled nitrogen-containing aza-crown ether (diazo-18-crown-6, DA18C6) into PA network interfacial polymerized by TMC and PEI. Figure 5c illustrated the cross-linked structure generated from amidation reaction and hydrogen bonding interactions among PEI, DA18C6, and TMC molecules. With abundant secondary amine groups that can provide reaction sites to generate stable covalent bonds to anchor on the surface or inside of the PA selective layer, the resulting PEI@DA18C6-PA membrane displayed a water permeance of 10.4 LMH/bar and a *S_Li,Mg_* of 11.2. Cyclodextrins and their derivatives possess a hydrophobic inner cavity coupled with a hydrophilic outer surface. Researchers have strategically introduced hydroxyl or amino groups to improve aqueous dispersibility, thereby reinforcing the membrane’s ion-sieving capability through pore size modulation [59,66,70]. β-CD@g-C_3_N_5_ was fabricated by Wang et al. [70] and incorporated it into the IP reaction between PEI and TMC. The membrane formed exhibited a Li^+^/Mg^2+^ selectivity coefficient of 38.85 and a permeability of 8.91 LMH/bar. Furthermore, studies indicate that rigid structural motifs and twisted polymer backbones can impede compact chain stacking, facilitating the formation of semi-permanent microcavities that establish extended continuous water permeation pathways [79]. A representative implementation involves Li et al. [66] embedding small-molecule AMN with stable chemical properties into PEI matrices. This approach increased interchain spacing within PEI networks, achieving 2.9-fold higher water flux compared to conventional PEI–TMC membranes.

The separation performance of nanofiltration membranes fabricated via IP can be optimized by regulating monomer dispersion in the bulk phase and stabilizing interfacial reaction kinetics. Surfactants containing distinct hydrophilic groups at both termini align at organic–aqueous interfaces, reducing water/organic interfacial tension and accelerating the diffusion of amine monomers from aqueous to hexane phases [80,81]. A representative demonstration by Peng et al. [75] achieved over 99.9% Mg^2+^ rejection through size-sieving mechanisms by incorporating oil-soluble surfactants (dodecyl phosphate, DDP) during TMC polymerization with PIP, as shown in Figure 5d. This process facilitates the formation of a thinner, more uniform polyamide active layer with narrower pore size distribution than conventional IP-derived PA layers [82,83,84].

### 3.3. Surface Modification

**Figure 6:**
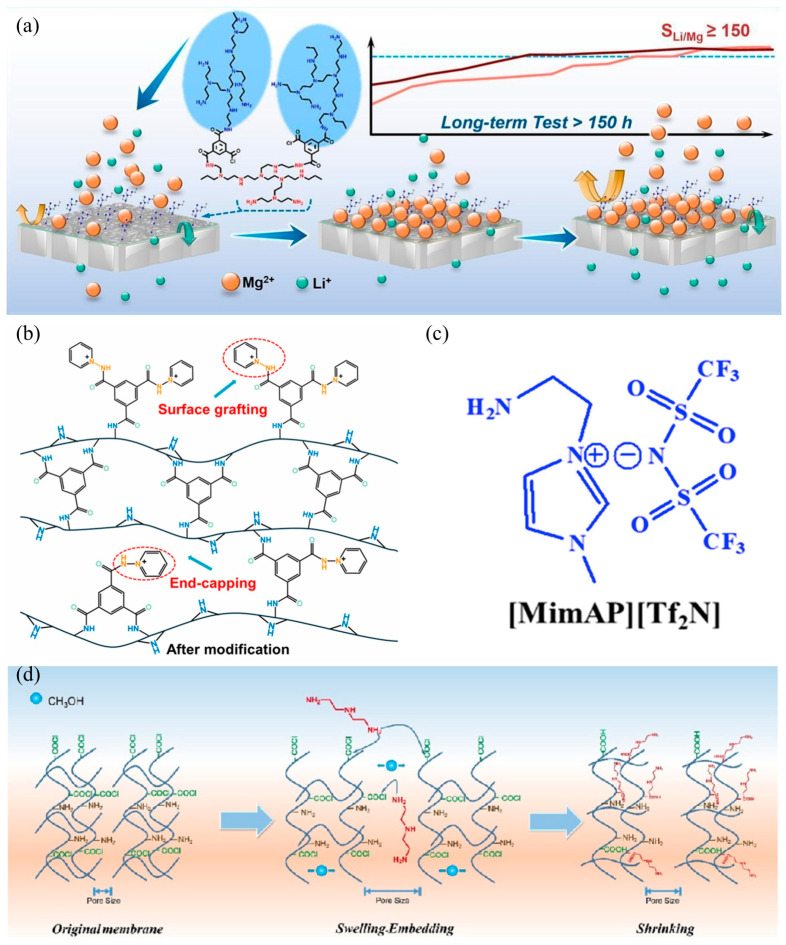
Schematic illustration of preparing lithium–magnesium separation membrane via surface modification: (**a**) preparation process of PEI-600/TMC/PEI-70000 nanofiltration membrane [88]; (**b**) capping–grafting regulation mechanism of 1-AI on the PEI separation layer [30]; (**c**) structure of [MimAP][Tf_2_N] [89]; (**d**) swelling—embedding—shrinking strategy [90].

Recent investigations have focused on post-processing strategies for nascent active layers, demonstrating that controlled post-treatments significantly modify membrane microstructure and surface physicochemical properties to enhance both permeability and Li^+^/Mg^2+^ selectivity [85,86]. Residual unreacted acyl chloride and carboxyl groups on pristine membrane surfaces typically limit positive charge density. These functional groups can be strategically grafted with charge-enhancing moieties, with representative studies shown in Table 3. Emerging studies reveal that grafting PEI directly onto NF membrane surfaces, rather than employing it as aqueous-phase monomer, reduces carboxyl group formation through reactions between PEI’s abundant amino groups and residual acyl chlorides. Concurrently, PEI’s amino groups amplify Donnan effects, intensifying positive surface charge [87]. As shown in Figure 6a, Peng et al. [88] employed PEI with varying molecular weights to prepare nanofiltration membranes through interfacial polymerization with TMC. They found that PEI (70,000 Da)-modified membranes demonstrated exceptional Li^+^/Mg^2+^ separation performance (*S_Li__,Mg_* = 150) when processing feed solutions with a Mg^2+^/Li^+^ ratio of 50; however, water flux remained suboptimal (2.18 LMH/bar). Complementary research by Lu et al. [85] revealed that low-molecular-weight PEI enables higher amino grafting density due to reduced steric hindrance, thereby improving membrane permeability.

**Table 3:** Representative membranes of positively-charged NF membrane for Mg^2+^/Li^+^ separation through surface modification.

Membrane	Mixture Concentration (ppm)	*R_Mg__2+_* (%)	*R_Li__+_* (%)	MLR	*PWP* (LMH/bar)	*S_Li,Mg_*	Ref.
PSf/PEI/TMC/QTHIM	2000	92.2	46.0	-	32.5	4.5	[91]
PSf/PEI/TMC/QBPD	2000	94.7	37.0	50	13.6	5.9	[92]
PAN/PIP/TMC/[MimAP][TFf_2_N]	2000	81.9	−45.2	20	6.3	8.1	[89]
PSf/PEI/TMC/HMTAB	2000	93.9	37.0	50	16.3	10.2	[93]
PES/PEI/TMC/TQAIL	10,500	92.8	26.6	-	22.2	10.5	[94]
PSf/PEI/TMC/DETA	-	93.9	31.0	24	2.8	11.4	[90]
PSf/PEI/TMC/PEI	2000	-	-	150	8.3	12.3	[85]
PES/PEI/TMC/DABIL	10,500	91.0	18.6	-	16.9	12.9	[95]
PSf/PEI/TMC/Cyclen	2000	86.3	50.7	20	1.3	15.3	[96]
PSf/PEI/TMC/QEDTP	2000	95.0	55.0	120	21.0	15.6	[28]
PES/PIP/TMC/ARG	2000	91.5	9.5	20	47.0	17.1	[97]
PES/PIP-HMAH/TMC/ATA	2000	95.7	18.1	20	9.4	19.2	[98]
PES/PIP/TMC/Am-CDs	2000	-	-	20	3.8	21.4	[99]
PSf/PEI/TMC/QTHEED	2000	94.3	−22.8	60	23.1	21.7	[100]
PSf/PEI/TMC/DCA	1000	96.0	37.0	20	10.9	23.3	[101]
PSf/PEI/TMC/1-AI	2000	97.1	-	20	19.8	27.7	[30]
PES/SWCNT-PDA/PIP/TMC/PEI	2000	98.5	46.2	20	7.4	35.9	[102]
PES/PEI/TMC/DHTAB	2000	95.4	65.4	-	6.7	58.0	[103]
PES, PSf/PA/G-AS-Fe	2000	86.6	22.7	20	55.7	81.5	[104]
PAN/PEI/TMC/BTPB	2000	98.9	30.6	-	~50.0	81.6	[105]
PAN/PEI/TMC/BTAB	2000	99.2	~30.0	20	50.0	95.9	[106]
PES/PEI/TMC/PEI	2000	99.5	-	50	2.1	150.0	[88]
PAN/PEI/TMC/TC	2000	99.1	30.6	20	~37.0	167.0	[107]
PSf/PEI/TMC/SBI	2000	91.0	46.4	100	16.0	/	[108]

The inherent characteristics of grafting monomers critically determine membrane performance. Beyond PEI, researchers have designed novel multi-charged monomers to engineer positively charged membranes with enhanced surface charge density. These innovative monomers include cationic polyelectrolytes, quaternary ammonium salts, or imidazolium-based ionic liquids, and typically incorporate positively charged functionalities such as amino groups, quaternary ammonium, or quaternary phosphonium moieties. Xu et al. [28] pioneered the use of a bis-quaternary ammonium monomer (QEDTP) for grafting via condensation reactions with residual acrylic acid groups on pristine PEI membranes, elevating the isoelectric point from 7.7 to 8.4. Zhao et al. [106] synthesized a novel quaternary ammonium bromide BTAB grafted onto PEI–TMC membranes through nucleophilic substitution. The modified membranes maintained water flux at about 10 LMH/bar while achieving 99.2% MgCl_2_ rejection and 70% LiCl permeation. Gu et al. [103] developed a multi-amino quaternary ammonium salt (DHTAB) grafted onto nascent membranes, attaining 99.2% MgCl_2_ rejection and Mg^2+^/Li^+^ separation factor of 60.1. These results underscore the significant potential of amino- or quaternary ammonium-functionalized monomers in constructing positively charged NF membranes. Li et al. [30] implemented 1-aminopyridinium iodide (1-AI) as a dual-functional agent—simultaneously acting as end-capper and grafting moiety during TMC interfacial polymerization. As shown in Figure 6b, this synergistic capping–grafting strategy enhanced membrane porosity, amplified surface positive charge density, and deepened grafting modification depth, collectively inducing substantial internal charge augmentation. The optimized membrane demonstrated superior performance with a water permeance of 19.8 LMH/bar and an *S_Li,Mg_* of 27.7.

Current grafting methodologies rely on pre-existing functional groups on membrane surfaces, rendering surface charge intensity constrained by the density of reactive sites and impeding precise modulation. Furthermore, existing research predominantly emphasizes enhancing surface positive charge magnitude while neglecting spatial charge uniformity. To address these limitations, researchers have developed two strategic approaches: (1) introducing additional active sites within PA layers, and (2) incorporating rigid structural motifs. These optimized membranes demonstrate improved pore size uniformity and charge distribution homogeneity, achieving a water permeance of 9.43 LMH/bar with a 95.74% MgCl_2_ rejection, 18.1% LiCl rejection, and salt selectivity of 19.22. Ren et al. [98] anchored hydroxyethyl methacrylate-hydrazide (HMAH) onto PA membrane surface to function as hydroxyl-active sites, which enabled acryloxyethyltrimethylammonium chloride (ATA) to be grafted. The modified membrane possessed a more uniform pore size and charge distribution and achieved a water permeance of 9.43 LMH/bar, MgCl_2_ rejection of 95.74%, LiCl rejection of 18.1%, and salt selectivity of 19.22. Zhang et al. [99] engineered amine-functionalized cyclodextrins (Am-CD) through sequential processes: first depositing on PES substrates, then grafting onto PIP-TMC membranes, ultimately achieving a Li^+^/Mg^2+^ selectivity of 21.4. Another innovative approach involves inserting rigid cationic monomers into flexible polyamide chains to remodel internal pore architecture and charge distribution at molecular scales—termed the “swelling—embedding—shrinkage strategy”. Li et al. [90] implemented this by methanol-swelling pristine PEI/TMC membranes to expand polyamide chain spacing, followed by embedding positively charged diethylenetriamine (DETA) to enhance charge density and modulate pore size (Figure 6d).

Conventional IP processes are fundamentally constrained by their reliance on water-soluble monomers reacting with acyl chlorides, which intrinsically limits monomer selection diversity. In addition, this aqueous/organic phase reaction mechanism inevitably exposes acyl chlorides to hydrolysis at the liquid–liquid interface, resulting in compromised PA layer crosslinking density and, consequently, diminished rejection performance in nanofiltration membranes. To address these limitations, ionic liquids (ILs) have emerged as innovative phase transfer catalysts [89,94,95]. Their unique solvation capabilities enable the effective dissolution of diverse amine monomers while providing an anhydrous environment for interfacial reactions with alkane-dissolved acyl chlorides, thereby establishing a novel IP platform that circumvents traditional hydrolysis challenges. Soyekwo et al. [95] engineered a novel cluster-structured multi-quaternary ammonium ionic liquid monomer (DABIL) that was covalently immobilized onto PEI–TMC NF membrane surfaces through sequential amidation reactions. The resultant DABIL-modified membrane demonstrated a water permeability of 16.9 LMH/bar and a Li^+^/Mg^2+^ selectivity of 26.49, which establishes a new paradigm for developing composite NF membranes with simultaneous high permselectivity and biofouling resistance.

Surface modification represents an effective approach for enhancing lithium–magnesium selectivity in membrane applications. While the grafting of quaternary ammonium salts or polyamino compounds strengthens surface positive charge density, precise control over membrane pore dimensions remains challenging. Prioritizing modifiers with high dissociation constants is essential to sustain protonation across a broad pH range, thereby optimizing ion separation performance in weakly alkaline salt-lake brine environments. Furthermore, the reliance on residual acyl chloride groups from initial interfacial polymerization introduces variability, as the stochastic distribution of these reactive sites compromises both reproducibility and long-term stability in membrane fabrication. This dual constraint of limited pore tunability and process-dependent grafting mechanisms underscores the need for innovative modification strategies to advance precision in membrane engineering.

### 3.4. Interlayer Integration

In recent years, a significant number of studies have shown that introducing an intermediate layer between the porous support layer and the separation layer can regulate the IP reaction and the microstructure of the PA layer, thereby enhancing the permeability and separation performance of thin-film composite membranes. Representative studies are shown in Table 4. The impact of the intermediate layer on the lithium–magnesium separation of nanofiltration membranes can be summarized in the following three aspects:

**Table 4:** Representative membranes of positively-charged NF membrane for Mg^2+^/Li^+^ separation through interlayer intergration.

Membrane	Mixture Concentration (ppm)	*R_Mg__2+_* (%)	*R_Li__+_* (%)	MLR	*PWP* (LMH/bar)	*S_Li,Mg_*	Ref.
PSF/ZIF-8/PA	2000	97.3	43.0	-	21.8	4.0	[109]
PSf/UIO-66-NH_2_/PA	2000	97.0	35.9	15.3	-	32.2	[110]
PES/zwitterion-g-C_3_N_4_/PA	2000	97.9	~30.0	48	9.1	37.8	[111]
PSf/CP8/PA	2000	98.5		13.9	8.1	39.0	[112]
PES/PIP/SDS/TMC	2000	96.8	37.8	-	11.0	42.1	[113]
PSf/160A/PA	2000	98.2	~25.0	7.5	-	45.8	[114]
PSf/polyphenol-PEI/PA	2000	93.6	28.8	-	18.6	50.7	[107]
PSf-NoriaPG-PEI/PA	2000	98.5	-	30.9	22.5	88.6	[115]
PSf/RA-PEI/PA	2000	93.6	28.8	20	16.7	92.8	[116]

**Figure 7:**
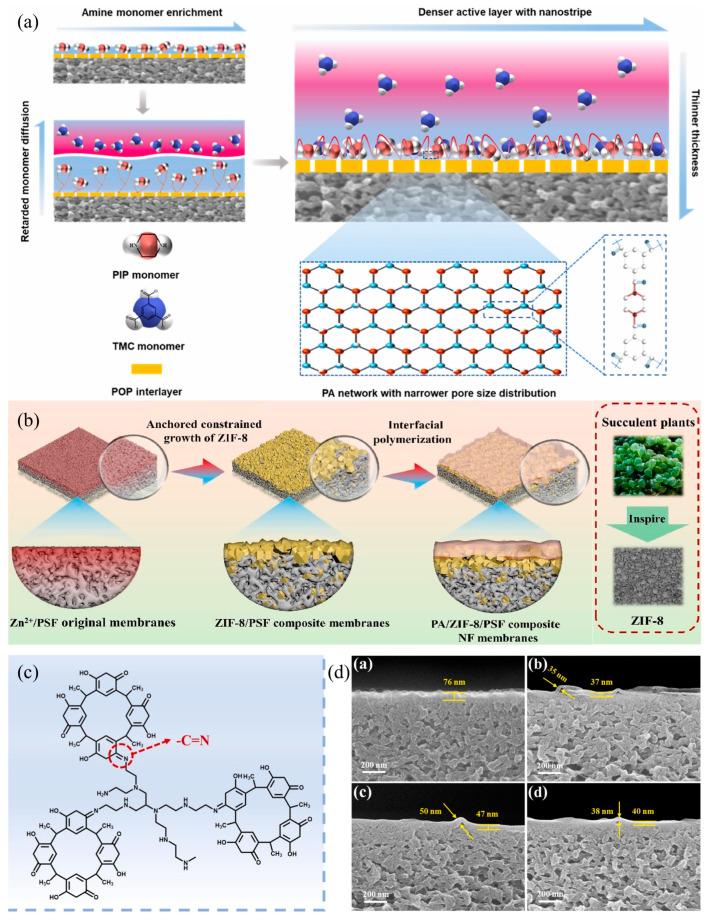
Schematic illustration of preparing lithium–magnesium separation membrane via interlayer intergration: (**a**) POP tailoring in the monomer adsorption and diffusion for the fabrication of PA active layers with thinner thickness and narrower pore size distribution [112]; (**b**) fabrication process for PA/ZIF-8/PSf composite NF membranes [109]; (**c**) reaction mechanism between PEI and RA [116]; (**d**) cross-sectional features of the PA/ZIF-8/PSf membranes:TFC-0, TFC-1, TFC-2, TFC-3 [121].

(i) Compared to traditional hydrophobic porous substrates, the hydrophilic interlayer is conducive to the adsorption of amine monomers at the interface, thereby increasing the storage of amines at the interface, making more amines available for IP. This is considered to minimize the formation of defects in the PA layer, thereby improving the rejection performance [117,118].
(ii) During the IP process, amine monomers will continuously diffuse from the aqueous phase to the organic phase. Therefore, the diffusion rate from the amine reservoir (i.e., interlayer or substrate) to the organic phase is crucial for the performance of the PA layer. Studies have shown that due to the interaction between the interlayer and the amine monomers (such as covalent bonds and hydrogen bonds), the interlayer exhibits a reduced amine desorption rate or slower amine diffusion [117,119]. The slow amine diffusion limits the available amine monomers that can enter the organic phase to react with TMC, thereby forming a thinner PA layer [120]. At the same time, it can also provide a more uniform pore structure for the IP process, which, in turn, makes the distribution of monomer solutions in the IP reaction more uniform. Many scholars have combined the intermediate layer with the base membrane through the electrophilic coupling reaction of diazonium salts to regulate the diffusion behavior of amine monomers in the oil phase, allowing them to react with acyl chlorides, thereby endowing the interfacial polymerization polyamide dense layer with optimized selective pore structure and reduced internal negative charge [110,112,114]. As shown in Figure 7a, Zhao et al. [112] introduced a double-rigid twisted (porous organic polymer) POP intermediate layer onto the base membrane, and the modified membrane has a narrow pore size distribution, a small average pore size, and a lithium–magnesium separation factor of up to 78.56.
(iii) In the magnesium–lithium separation system, due to the synergistic effect between size sieving and Donnan equilibrium, compared to the negatively charged intermediate layer, the positively charged intermediate layer can endow the nanofiltration membrane with appropriate pore size and surface charge density, thereby achieving higher Mg^2+^/Li^+^ separation selectivity [115]. This is mainly because there is no strong interaction between the positively charged intermediate layer and the amine monomer, thus allowing for the rapid release of a large number of amine monomers from the intermediate layer. These amine monomers diffuse into the IP reaction zone and react with TMC to form a dense PA layer with a smaller average pore size and a narrower pore size distribution. Conversely, for the negatively charged intermediate layer, the strong electrostatic interaction between the amine monomer and the intermediate layer inhibits the release of amine monomers from the intermediate layer, slowing down the diffusion of amine monomers to the IP reaction zone, resulting in a more porous PA layer for the negatively charged intermediate layer nanofiltration membrane. For example, Chen et al. [121] used catechol (CA), hydroquinone (HQ), and pyrogallol (PG) to crosslink PEI to form nanoscale aggregates on a PSf substrate to prepare a positively charged hydrophilic polyphenol intermediate layer, and by controlling the distribution and diffusion of PIP, a highly crosslinked PA layer was formed. Compared to the original membrane (TFC-0), the modified membrane’s PA layer thickness is reduced (35-50 nm), the average pore size is smaller (as shown in Figure 7d), the rejection rate for Mg^2+^ is higher, and the lithium–magnesium separation factor is 50.7.

To address the issue of poor adhesion between the intermediate layer and the support membrane, studies have found that the PA/interlayer adhesion force is similar in many aspects to the adhesion force between the interlayer and the base membrane, as both the PA layer and typical polymer base membranes possess oxygen-containing functional groups and conjugated structures [118]. When an intermediate layer is introduced between the porous substrate and the PA layer, the adhesion force between the interlayer/base membrane and the PA/interlayer changes accordingly, which mainly depends on the type of the intermediate layer. Typically, organic intermediate layers are rich in functional groups that can interact with the polymer base membrane through electrostatic interactions, hydrogen bonds, Van Der Waals forces, π-π conjugation, and covalent bonds, resulting in a tight adhesion between the organic interlayer and the base membrane, forming a more uniform intermediate layer and enhancing the stability of the PA layer [122,123,124]. In recent years, many scholars have used the principle mentioned above, employing co-deposition methods to modify the base membrane, and have obtained nanofiltration membranes with higher lithium–magnesium separation performance. Bai et al. [116] deposited the C-Methylcalix resorcinarene (RA)-PEI interlayer on the surface of the PSf substrate with firm adherence due to the synergistic effect of intermolecular hydrogen bonding and π-π interactions (as shown in Figure 7c). This endowed the membrane a narrower pore size distribution, smaller average pore size, and higher positive charge. In salt mixture tests, the modified membranes exhibit a higher lithium–magnesium separation factor (*S_Li,Mg_* = 92.8) and permeability (*PWP* = 16.7 LMH/bar). In addition, inspired by the controlled growth mechanisms of succulent plants, Tian et al. [109] pre-anchored zinc ion ligands of ZIF-8 onto the PSf substrate to provide reactive sites, enabling the growth of ZIF-8 nanoparticles embedded within the PSf surface layer to fabricate a positively charged PA/ZIF-8/PSf composite NF membrane. Figure 7b illustrates the fabrication process, and this structural integration enhanced interfacial bonding strength between the PSf support and ZIF-8 interlayer, achieving improved membrane permeability (18.7 LMH/bar) while maintaining over 98% of Mg^2+^ rejection at 5 bar operating pressure.

Additional studies have shown that Janus membranes composed of a negatively charged intermediate layer and a positively charged PA layer also exhibit excellent lithium–magnesium separation performance, which seems to contradict the results we have discussed, and further research and exploration will be needed in the future [113,125]. Some scholars believe that 2D nanomaterial intermediate membranes have significant advantages such as large lateral dimensions, high aspect ratios, and ultra-thin thicknesses, which can form tortuous permeation channels, thus more effectively enhancing selectivity [126]. In summary, in order to better develop high-separation-performance nanofiltration membranes that incorporate an intermediate layer, it is necessary to deeply reveal the influence mechanism of the interlayer structure on the PA layer, and to further explore materials for constructing the intermediate layer.

### 3.5. Substrate Functionalization

In addition to serving as mechanical support, the physical structural characteristics and chemical group properties of the microporous substrate, including chemical reactivity, hydrophilicity, and porosity, play a significant role in the formation of the PA separation layer, which can greatly affect the separation selectivity of the composite NF membrane for monovalent/divalent ions [127,128,129,130,131]. During the IP process, the aqueous phase generally wets the structure of the support substrate first, and then diffuses into the oil phase monomer for the polycondensation reaction. Therefore, the physical structure of the substrate (pore size and porosity) will determine the storage capacity of the amine monomers, while its chemical properties will determine the diffusion rate of the amine monomers, thereby affecting the formation of the polyamide structure and, ultimately, the performance of the TFC membrane. Compared to the strategy of introducing an intermediate layer as described in Section 3.4, substrate modification directly involves physical or chemical treatment of the substrate, usually aimed at enhancing the permeability, mechanical strength, or compatibility with the separation layer of the substrate. Methods for substrate modification typically include three approaches: (i) substrate surface coating, such as applying a layer of PDA, GO, chitosan, etc., onto the substrate surface; (ii) grafting functional molecules or polymer chains onto the substrate surface through chemical reactions; and (iii) adding nanomaterials to the substrate casting solution. Representative studies are shown in Table 5.

**Figure 8:**
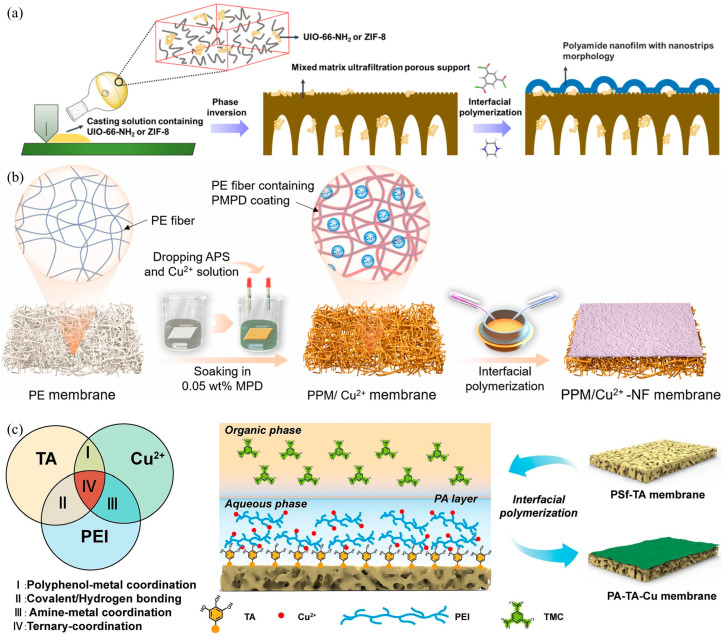
Schematic illustration of preparing lithium–magnesium separation membrane via substrate functionalization: (**a**) fabrication process of the nanostriped PA nanofilm atop the MMMs [138]; (**b**) hydrophilization of PE substrate and the fabrication of the PE-based NF membrane [137]; (**c**) ternary coordination among TA, PEI, and Cu^2+^ ions and IP on TA-modified substrate (PSf-TA membrane) based on the polyphenol-metal-assisted method [136].

As we have discussed in Section 3.2, nanomaterials with tunable properties have been extensively added into the PA active layer. Wei et al. [139] discussed the impact of incorporating nanomaterials at different positions in composite membranes on the membrane performance. They found that the incorporation of hydrophilic nanomaterials can increase the hydrophilicity and wettability of the substrate and also improve the thermodynamic incompatibility between the polymer and the solvent. In recent years, many scholars have mixed one-dimensional carbon nanotubes [140], two-dimensional MOF [109,138], MXene [132], and GO nanosheets [133,135], and other nanomaterials into the substrate. For example, UIO-66-NH_2_ or ZIF-8 was doped with mixed matrix UF membranes (MMMs) to fabricate PA membranes by Yuan et al. [138], which endows the membrane with fine-tuning structure in surface charge and inner pore size (Figure 8a). With porous coordination, the fabricated nanofilm with ZIF-8 doped UF substrate showed a Li^+^/Mg^2+^ selectivity of 377.8 and 2.18–2.67 times enhancement of water flux. However, the inherent characteristics of nanomaterials still present defects in the modification of the base film, such as an increase in viscosity of the casting solution containing an excessive amount of nanomaterials [141], and severe aggregation or deposition of nanomaterials due to the incompatibility between nanomaterials and polymers [142]. These issues can reduce the chemical and physical stability of the substrate and hinder the formation of a defect-free PA layer.

Conventional studies combining the physical adsorption of different charges between PEI and negatively charged PES and PSf UF substrates without chemical interaction often lead to a weak adhesion between the support layer and the active layer. To tackle this issue, researchers attempted to limit the diffusion rate of monomers by constructing a ternary synergistic effect of substrate–monomer–salt ions, thereby forming a modified membrane with a thinner thickness, a looser structure, and more uniform pore sizes. Poly(m-phenylenediamine) (PMPD) coating on PE fibers was first grown in situ and then coordinated with Cu ions by Wen et al. [137], as shown in Figure 8b. Cu^2+^ forms a chelation with the abundant amino groups in PMPD and interacts with PEI to synergistically regulate the diffusion of PEI into the organic phase. In a simulated brine experiment with a lithium-to-magnesium ratio of 20, the modified membrane’s permeability reached 16 LMH/bar, and the lithium–magnesium separation factor reached 33. Fang et al. [136] established a ternary interaction among PEI, Tannic acid (TA), and Cu ions, by first depositing TA on the substrate and then immersing it in a Cu^2+^-doped PEI monomer aqueous solution. The separation mechanism is illustrated in Figure 8c. The modified membrane exhibited a relatively high Mg^2+^ rejection rate (95.9%) and a low LiCl rejection (17.1%), with a pure water permeance of 4.87 LMH/bar and separation factor of 26.5.

The physicochemical structure and properties of the support layer and intermediate layer are crucial for the construction process of positively charged composite nanofiltration membranes, but there is still limited research on this topic. How to optimize the properties of the support layer and intermediate layer to achieve precise control and charge compensation of the separation layer structure of positively charged composite NF membranes, thereby enhancing the permeability selectivity and stability of the membranes, requires further exploration.

## 4. Fundamental Characteristics of NF Membrane for Mg^2+^/Li^+^ Separation

The solute rejection and permeation performance of NF membranes are governed by both the external operating parameters as well as intrinsic membrane properties. On the one hand, external factors such as pH, pressure, temperature, and MLR affecting Mg^2+^/Li^+^ separation have been extensively discussed, showing that operating conditions can significantly influence lithium–magnesium separation performance [143,144]. On the other hand, the performance of membranes also depends on their structure and physicochemical characteristics such as pore size and distribution, thickness of the separation layer, surface charge characteristics, hydrophilicity, and roughness. Understanding the membrane performance–structure relationships and how manufacturing methods and conditions affect membrane performance will help in tailoring the design of NF membranes for Mg^2+^/Li^+^ separation [145]. Therefore, this section primarily summarizes the impact of internal structural factors, aiming to provide a reference for the structure–performance relationship of positively charged NF membranes for Mg^2+^/Li^+^ separation.

### 4.1. Pore Size and Distribution

In lithium–magnesium separation, the precise regulation of polyamide active layer pore size stands as a critical determinant of separation efficiency in positively charged NF membranes. According to size sieving theory, complete solute rejection occurs when hydrated ion radii exceed membrane pore dimensions. However, structural defects and pore heterogeneity in fabricated active layers compromise separation selectivity, driving research toward developing isoporous NF membranes. Key parameters governing pore size regulation through IP processes include (i) monomer chemical characteristics, for example, employing acyl chloride monomers with compact molecular conformations creates densely crosslinked structures, with molecular dynamics simulations demonstrating effective pore sizes below 0.6 nm; and (ii) reaction kinetic control, i.e., increasing aqueous monomer concentration, optimizing reaction temperature, and adjusting pH accelerate monomer diffusion rates to promote crosslinked network formation.

Studies reveal that pore size regulation faces the inherent selectivity–permeability trade-off dilemma. While smaller pore sizes may enhance selectivity, they typically induce significant permeability reduction, whereas larger pores compromise solute rejection. Consequently, researchers have developed rigorous mathematical models that holistically incorporate multiple parameters—including pore size, effective membrane thickness, and average charge density—rather than isolating pore dimension effects [146,147,148,149]. These models have been successfully employed to describe the relationships between membrane structural/physicochemical properties and solute rejection performance.

The pore size distribution of membranes significantly influences rejection selectivity or separation precision, where enhanced pore uniformity benefits lithium–magnesium separation efficiency. For NF membranes fabricated via IP, the polyamide active layer exhibits structural heterogeneity in both cross-sectional and planar orientations, featuring non-uniform pore sizes due to substrate membrane morphology, chemical properties, and inherent IP process variability. Strategies such as improving substrate hydrophilicity, constructing interlayer structures, or incorporating two-dimensional materials can promote uniform amine monomer distribution, effectively reducing defect formation and facilitating a more homogeneous active layer architecture.

### 4.2. Zeta Potential

In addition to pore size, surface charge intensity of the membrane also serves as a critical parameter, directly reflecting membrane surface charge properties determined by the dissociation states of interfacial chemical groups. The dynamic coupling between membrane surface zeta potential and solution pH centers around the isoelectric point (IEP), which means surfaces exhibit positive charge when pH < IEP and negative charge when pH > IEP. While commercial NF membranes typically employ negative charge modification to enhance biofouling resistance [150], this conflicts with lithium–magnesium separation requirements: Maintaining surface positivity within operational pH ranges is essential to achieve high Mg^2+^/Li^+^ separation factors through Donnan exclusion effects (theoretical Mg^2+^ rejection exceeding 95%) [151]. Therefore, the optimization objective involves elevating membrane IEP significantly above the feed solution pH.

As we discussed before, to balance ideal zeta potential requirements in practical engineering applications, three strategic recommendations are proposed: (i) establishing pH-zeta potential response databases tailored to specific brine system characteristics; (ii) adopting multilayer composite architectures incorporating charge-buffering interlayers beneath active layers; and (iii) developing pH-responsive smart membrane materials enabling dynamic charge regulation.

### 4.3. Hydrophilicity

Many scholars are dedicated to improving the hydrophilicity of nanofiltration membranes when separating lithium and magnesium ions. This might be by utilizing strongly hydrophilic surfaces that could facilitate hydration layer formation, reducing liquid permeation resistance, thereby improving water flux. According to the Wenzel model, rough surfaces can amplify the wettability of hydrophilic materials, further reducing the contact angle and enhancing the formation of hydration layers, thereby indirectly improving separation performance. On one hand, increased effective permeation surface area [152] reduces mass transfer resistance and mitigates concentration polarization. On the other hand, when combined with hydrophilic material modification, rough surfaces can establish hierarchical wetting structures that amplify membrane hydrophilicity, thereby enhancing pure water permeability (*PWP*).

Researchers have made substantial efforts to construct rough surface structures for expanding active layer surface area and improving water flux, employing strategies such as composite coating, co-monomer modification, and nanomaterial doping. Feng et al. [153] introduced a QBPD layer to create micro-nano wrinkles, elevating surface roughness (Ra) from 15.2 nm to 68.5 nm while simultaneously improving hydrophilicity and *PWP*. Notably, while conventional IP-based PA layers derive mechanical strength from substrate micropore anchoring, surface wrinkling treatments reduce the contact area between the active layer and substrate. This reduction compromises interfacial adhesion strength and mechanical stability.

While hydrophilicity improvement significantly boosts water permeability, it may deplete surface functional groups, causing zeta potential shifts that compromise Donnan exclusion effects. These approaches variably influence solute rejection, depending on whether they reduce active layer crosslinking density. For lithium–magnesium separation systems, carboxyl-amine bulk modification is prioritized to balance hydrophilicity enhancement with sustained separation performance.

### 4.4. Thickness of the Separation Layer

Apart from the aforementioned membrane surface characteristics, the thickness of the active layer also has a significant impact. During lithium–magnesium separation using positively charged NF membranes, the active layer thickness governs separation performance through multiple mechanisms. (i) Mass transfer kinetics: When water molecules traverse the active layer into microporous substrates, deviations of permeation paths from pore axes generate significant mass transfer resistance [154], causing permeate flux attenuation. (ii) Selectivity regulation: Since, in general, the pores are not perpendicular to the membrane surface, the average permeation path through the PA layer will be longer than the thickness of the PA layer. Reducing the active layer thickness (typically <100 nm) enhances Mg^2+^ rejection by shortening Mg^2+^ migration pathways and intensifying charge repulsion effects. (iii) Mechanical stability: Ultrathin active layers (<100 nm) risk structural failure or deformation due to insufficient mechanical strength, exacerbating membrane fouling and performance degradation. (iiii) Energy efficiency: Thin-layer architectures enable high-flux operation under low pressure, effectively reducing energy consumption per unit water production.

In traditional IP processes, the high hydrophobicity, low porosity, and surface roughness of substrate membranes often lead to uneven amine monomer distribution, requiring extended reaction times at low monomer concentrations to form continuous defect-free active layers. The Livingston team [26] pioneered an interlayer construction strategy by introducing transition layers between substrates and active layers for structural optimization. This field has evolved two predominant material systems. (i) Organic systems including PDA/TA/Fe^3+^ complexes and PVA, which exhibit exceptional interfacial adhesion and process adaptability. Their strong hydrophilicity creates “water channel” effects, analogous to the “gutter” mechanism in gas separation membranes [155], effectively reducing lateral mass transfer resistance. (ii) Inorganic nanomaterial systems such as CNTs, cellulose nanocrystal/MXene hybrids, COFs, and MOFs, which leverage high porosity to reduce active layer thickness to nanoscale. However, their larger surface pore structures may generate localized defects under high pressure, causing rejection rate fluctuations.

Building upon the Freger–Srebnik theoretical model [156], which establishes a cubic root proportionality between active layer thickness and amine monomer diffusion rate, recent studies have unveiled two effective diffusion regulation strategies: (i) interlayer synergy, where enhanced anchoring of PIP molecules through intermolecular interactions (e.g., electrostatic attraction and hydrogen bonding) retards their diffusion toward the organic phase; and (ii) the glycerol additive strategy, where precise control of active layer thickness is achieved through aqueous phase viscosity modulation via glycerol incorporation, coupled with long-range electrostatic interactions from phosphate-modified substrates.

In summary, while active layer thinning significantly reduces mass transfer resistance and enhances water permeability, it compromises the acid/base/oxidant resistance of TFC membranes, posing challenges for industrial applications requiring frequent chemical cleaning. A comprehensive evaluation system should be established through (i) the mechanical integrity testing of active layers; (ii) long-term operational stability assessments; and (iii) the analysis of the feed channel architecture’s impact on membrane surface shear stress. This multi-dimensional framework will provide theoretical foundations for membrane module design and process optimization.

## 5. Summary and Outlooks

The rapid evolution of lithium extraction technologies has positioned positively charged NF membranes as a transformative solution for addressing the global demand for high-purity lithium, particularly in salt lake brines with high Mg^2+^/Li^+^ ratios. This review comprehensively examines the design and fabrication strategies of IP-based positively charged NF membranes, emphasizing their superior performance in lithium–magnesium separation through synergistic size sieving and enhanced Donnan exclusion effects. By categorizing advancements into five key approaches—monomer engineering, additive incorporation, surface modification, interlayer integration, and substrate functionalization—this work elucidates how structural parameters such as pore size uniformity, surface charge density, hydrophilicity and active layer thickness critically govern separation efficiency. Innovations in PA layer design, including Janus architectures, crown ether-functionalized channels, and quaternary ammonium grafting, have demonstrated remarkable improvements in selectivity while maintaining competitive water permeability. Furthermore, the integration of nanomaterials and ionic liquids has enabled precise control over membrane morphology and charge distribution, effectively mitigating the permeability–selectivity trade-off. In the quest for optimal positively charged NF membranes for Mg^2+^/Li^+^ separation, various strategies have been explored. Among them, membranes incorporating Janus architectures or functionalized with crown ethers have shown remarkable selectivity improvements. For instance, the Janus membrane fabricated by Qi et al. [33] achieved a high *S_Li,Mg_* of 24.0, while He et al.’s crown ether-functionalized membrane exhibited a retention rate of 97.3% for magnesium ions [34]. In terms of *PWP*, membranes modified with certain ionic liquids or nanomaterials like g-C_3_N_5_ have demonstrated notable performance, with some achieving *PWP* values exceeding 50 LMH/bar [64]. The most suitable membranes balance high selectivity with sufficient permeability and stability. For example, the PSf/EDA@PEI/TMC membrane achieved a maximum Mg^2+^ rejection rate of 99.2% and an *S_Li,Mg_* of 80.6, while maintaining a *PWP* of 5.2 LMH/bar [31].

Despite these advancements, how manufacturing process routes affect the long-term stability or antifouling performance of membranes in harsh saline environments, as well as how membrane performance metrics compare across different systems, remain issues requiring future research. The manufacturing process significantly influences the membrane’s long-term stability and fouling resistance in harsh brine environments. Traditional interfacial polymerization methods often result in dense but potentially brittle polyamide layers, susceptible to hydrolysis and mechanical degradation under prolonged exposure to high salinity and pressure. Strategies such as interlayer integration and substrate functionalization can enhance membrane durability by providing additional structural support and reducing defect formation. Moreover, incorporating hydrophilic nanomaterials or modifying the membrane surface with zwitterionic compounds can improve antifouling properties by minimizing organic and inorganic deposition. Future advancements should focus on optimizing these processes to ensure robustness and reliability in industrial applications. Future research should prioritize enhancing membrane stability, focusing on the development of chlorine-resistant membranes, pH-responsive charge modulation systems, and standardized evaluation protocols to bridge laboratory-scale innovations with large-scale applications. To compare membrane performance across different systems, key metrics such as *S_Li,Mg_*, *PWP*, and rejection rates must be standardized. For instance, membranes with higher *S_Li,Mg_* values indicate superior selectivity, while higher *PWP* signifies better permeability. Rejection rates for Mg^2+^ and Li^+^ should be evaluated under consistent conditions to ensure comparability. Additionally, the trade-off between selectivity and permeability should be carefully considered. Membranes achieving a balance with high *S_Li,Mg_* and moderate *PWP* are often more practical for industrial applications. Comparisons should also account for operational conditions such as pH, pressure, and temperature, as these factors significantly influence membrane performance. By addressing these gaps—through focused material design, surface engineering, and rigorous testing—the next generation of NF membranes could revolutionize lithium extraction, aligning with sustainable energy transition goals and reducing reliance on conventional resource-intensive methods.

## Figures and Tables

**Table 1:** Representative membranes of positively-charged NF membrane for Mg^2+^/Li^+^ separation through monomer engineering.

Membrane	Mixture Concentration (ppm) ^1^	*R_Mg__2+_* (%) ^2^	*R_Li__+_* (%) ^3^	MLR ^4^	*PWP* (LMH/bar) ^5^	*S* *_Li,Mg_*	Ref.
PAN/DAPP/TMC	2000	70.4	21.8	20	2.6–2.8	2.6	[20]
PSF/QSPIP/TMC	2000	~93.0	~30.0	40	~20.0	~8.7	[37]
PSF/GEM/TMC	2000	94.8	40.0	100	17.5	13.1	[38]
PES/1,3-diaminoguanidine-PEI/TMC	2000	96.7	62.7	20	22.4	13.6	[39]
PSF@PDA/PEI/TBB	2000	94.7	42.8	20	4.2	16.7	[40]
PES/PEI/TMC	2000	94.8	30.6	20	5.0	20.0	[25]
PSF/PEI@GRT/TMC	2000	97.2	-	20	115.0	22.7	[32]
PES/DAGH/TMC	2500	95.0	36.1	10	12.2	23.3	[41]
PSF/PIP-PEI/TMC	2100	98.5	21.0	20	16.0	24.0	[33]
PSF/TET/TMC	2000	95.5	27.0	50	18.0	28.0	[29]
PES/PEI/TPC	2000	>97.0	<38.0	20	4.8	30.9	[42]
PAN/TG/TMC	2000	98.7	59.2	120	3.0	36.4	[43]
PSF/PIP/TMC	2000	>99.0	<11.3	20	6.8	45.3	[36]
PES/QBPIP/TMC	2000	98.8	12.3	31.2	28.3	76.9	[44]
PSF/EDA@PEI/TMC	2000	99.2	36.7	60	5.2	80.6	[31]
PK/PAA/IPC	2000	99.1	23.3	20	7.4	82.8	[45]
PSf/PIP, HACC/TMC	2000	94.3	−4.0	13.9	15.7	115.0	[46]
PK/PAA/TMC-IPC	2000	99.3	<55.0	20	9.3	117.0	[35]

^1^ The mixture concentration refers to mixed solution of MgCl_2_ and LiCl if there is no explanation. ^2^ *R_Mg2+_* (%) refers to the rejection rate of magnesium ions. ^3^ *R_Li+_* (%) refers to the rejection rate of lithium ions. ^4^ MLR refers to the Mg^2+^/Li^+^ mass ratio of the feed solution. ^5^ LMH/bar refers to L/m^2^·h·bar.

**Table 2:** Representative membranes of positively-charged NF membrane for Mg^2+^/Li^+^ separation through additive incorporation.

Monomers and Additives into PA	Substrate Membrane	Mixture Concentration (ppm)	*R_Mg__2+_* (%)	*R_Li__+_* (%)	MLR	*PWP* (LMH/bar)	*S_Li,Mg_*	Ref.
PEI/TMC+Cyclen	PSf	2000	90.4	22.0	20	14.0	8.7	[58]
PEI/TMC+γ-CDs	PES	2000	96.0	-	30	4.8	10.8	[59]
PEI/TMC+DA18C6	PSf	2000	96.3	43.4	20	10.4	11.2	[60]
PIP/TMC+PHF	PES	2000	89.9	16.3	21.4	6.7	13.1	[61]
PEI/TMC+PDA@BNNSs-NH_2_	PSf	2000	94.0	<35.0	75	8.5	15.6	[62]
PEI/TMC+TiO_2_-NH_2_	PES	2000	94.6	-	20	57.9	16.3	[63]
PEI/TMC+g-C_3_N_5_	PES	2000	94.5	32.8	20	58.6	18.2	[64]
BAPP/TMC+g-C_3_N_4_@MBCN	PES	2000	97.4	-	73	-	23.9	[65]
PEI/TMC+AMN	PES	2000	91.8	32.1	50	13.0	26.7	[66]
PEI+ZIF-8-NH_2_/TMC	MCE	2000	91.3	23.7	-	-	33.1	[67]
PEI/TMC+UiO-66-NH_2_	PAN	2000	97.4	4.1	20	30.6	36.9	[68]
PIP/TMC+F-SiO_2_	PES	2000	95.7	−63.2	20	56.0	37.9	[69]
PEI/TMC+β-CD@g-C_3_N_5_	PES	2000	~97.8	~30.0	20	8.9	38.5	[70]
PEI/TMC+MWCNTs-COOK	PES	2000	98.6	21.6	20	12.2	58.0	[71]
BAPP/TMC+g-C_3_N_4_	PES	2000	96.1	42.3	73	17.0	102.0	[72]

**Table 5:** Representative membranes of positively-charged NF membrane for Mg^2+^/Li^+^ separation through substrate functionalization.

Membrane	Mixture Concentration (ppm)	*R_Mg__2+_* (%)	*R_Li__+_* (%)	MLR	*PWP* (LMH/bar)	*S_Li,Mg_*	Ref.
PES-MXene/PEI/TMC	2000	89.7	21.4	-	16.1	15.8	[132]
PEI/TMC/CNC-COOK	2000	95.1	20.9	20	11.1	16.1	[133]
PES@γ-CD/TPC/PEI	2000	>90.0	<25.0	30	6.5	22.5	[134]
PES/GO/PA	-	95.9	67.6	23	-	23.5	[135]
PSf-CuCl_2_/PEI/TMC	2000	96.0	−7.1	-	4.8	26.5	[136]
PE-Cu^2+^/PEI/TMC	2000	~98.0	39.1	-	16.0	33.0	[137]
PSf-ZIF-8/PEI/TMC	2000	97.3	43.0	-	47.2	47.6	[109]
PES@MWCNTs-COOK/PEI/TMC	2000	98.6	21.5	20	12.2	58.6	[71]
PSf@UiO-66-NH_2_/PIP/TMC	2000	97.9	−66.7	30.6	50.2	78.6	[138]

## Data Availability

Data will be made available on request.

## Abbreviations

The following abbreviations are used in this manuscript:

[MimAP][TFf_2_N]	amine-functionalized ionic liquid 1-(3-aminopropyl)-3-methylimidazolium bis(trifluoromethanesulfonyl)imide
160A	160-Aromatic-NH_2_
1-AI	1-Aminopyridinium iodide
Am-CDs	amine-functionalized carbon dots
AMN	aminomalononitrile
ARG	arginine monohydrochloride
ATA	acryloxyethyl trimethyl ammonium chloride
BNNS	boron nitride nanosheets
BTAB	3-bromopropyl trimethylammonium bromide
BTPB	3-bromopropyl triphenyl phosphonium bromide
CDs	cyclodextrins
CNC-COOK	carboxylated cellulose nanocrystal
CP8	The PSf substrates modified using TTSBI/BAPF interlayers in which C and 8 represented cross-link and the pH values of the diazonium reagent solution.
Cyclen	1,4,7,10-tetraazacyclododecane
DA18C6	diazo-18-crown-6
DABIL	N1-(6-aminohexyl)-N1,N1,N6,N6,N6-pentamethylhexane-1,6-diaminium bromide
DAGH	1,3-diaminoguanidine hydrochloride
DAPP	1,4-bis(3-aminopropyl) piperazine
DCA	4,7,10-Trioxygen-1,13-tridecanediamine
DDP	dodecyl phosphate
DETA	diethylenetriamine
DHTAB	3,5-dimethylhydrazine-benzyltrimethylammonium bromide
EDA	ethylenediamine
EDTA	ethylenediaminetetraacetic acid
g-C_3_N_4_	nano graphitic carbon nitride
g-C_3_N_5_	amine-rich graphitic carbon nitride
GEM	Gemini electrolyte monomer
GO	graphene oxide
GRT	Girard’s Reagent T
HACC	hydroxypropyltrimethyl ammonium chloride chitosan
HMAH	3-[n-tris(hydroxymethyl)methylamino]—2-hydroxypropanesulfonic
HMTAB	1-(2-hydroxyethyl)-1,3,5,7-tetraazaadamantane-1-ium bromide
IPC	isophthaloyl chloride
MWCNTs-COOK	potassium carboxylate functionalized multi-wall carbon nanotubes
PA	polyamide
PAA	polyallylamine
PAN	Polyacrylonitrile
PDA	polydopamine
PE	polyethylene
PEI	polyethyleneimine
PES	polyethersulfone
PHF	polyhydroxylated fullerene
PIP	piperazine
PSf	polysulfone
QBPD	quaternized bipyridine
QBPIP	quaternized-bis piperazine
QEDTP	quaternized N, N, N’, N’-tetrakis (2-hydroxypropyl) ethylenediamine
QSPIP	quaternized-spiral piperazine
QTHIM	quaternized tetrahydroxyethyl imidazolium
SBI	spirocyclic diamine
SDS	sodium dodecyl sulfate
TBB	1,3,5-tris(bromoethyl)benzene
TC	2, 3-epoxypropyl trimethyl ammonium chloride
TET	trimesoyl chloride
TG	triaminoguanidine
TMC	trimesoyl chloride
TQAIL	triple-quaternary ammonium based ionic liquid
β-CD	β-Cyclodextrin
γ-CDs	γ-cyclodextrins
